# Dietary restriction in obese children and its relation with eating behavior, fibroblast growth factor 21 and leptin: a prospective clinical intervention study

**DOI:** 10.1186/s12986-015-0027-0

**Published:** 2015-09-15

**Authors:** Lorena del Rocío Ibarra-Reynoso, Liudmila Pisarchyk, Elva Leticia Pérez-Luque, Ma. Eugenia Garay-Sevilla, Juan Manuel Malacara

**Affiliations:** Department of Medical Sciences, University of Guanajuato, Campus León. 20 de Enero 929. Col Obregón, León Gto, México 37320

## Abstract

**Background:**

Obesity is significant problem involving eating behavior and peripheral metabolic conditions. The effect of carbohydrate and fat restriction on appetite regulation, fibroblast growth factor 21 (FGF21) and leptin in children has not been defined. Our objective was to compare the effect of both diets.

**Methods:**

One hundred and twenty children with body mass index (BMI) higher than the equivalent of 30 kg/m^2^ for an adult, as corrected for gender and age were randomly assigned to (*n* = 60) a low-carbohydrate (L-CHO) diet or (*n* = 60) a low-fat (L-F) diet for 2 months. Fifty-three (88.3 %) subjects on the low-carbohydrate-diet and 45 (75 %) on the low-fat diet completed the study. Anthropometric measures, leptin and FGF21 levels were measured before and after the intervention. Comparison of the data for both of the diet groups was carried out using the t-test for independent variables. Intragroup comparisons before and after of each of the dietary treatments were performed using ANOVA for repeated measures. Factors associated with FGF21, leptin levels and satiety, were analyzed by multiple regression.

**Results:**

After both of the diets, weight, leptin, food responsiveness, and enjoyment of food significantly decreased and high density lipoprotein cholesterol (HDL) increased, but FGF21 decreased. Before and after both of the interventions FGF21 was associated with triglycerides. Before the diet, satiety was associated with lower screen time (*p* < 0.04) and insulin levels (*p* < 0.05).

**Conclusions:**

Both dietary restrictions improved the metabolic and hormonal parameters of obese children. FGF21 is an indicator of a beneficial metabolic response in younger children. After 2 months an adaptation of the eating behavior to food restriction was observed.

## Background

Childhood obesity is a health problem that is difficult to manage because the obesogenic environment hinders long-term adherence to a diet. Eating behavior and energetic balance are controlled by neural circuits in the hypothalamic and hindbrain centers, which have afferent pathways related to sensory and hedonic centers, metabolic hormones and nutrient and adiposity signals [1].

Several programs of caloric restriction have been designed. Among them, carbohydrate or lipid restrictions have been used extensively with limited long-term results. In children, carbohydrate restriction offers a satisfactory weight loss and improvement of adiponectin levels [2]. Lipid restriction may improve cardiometabolic risk factors such as triglycerides, low density lipoprotein cholesterol (LDL) and insulin levels [3]. Lipid restriction may also improve Homeostatic Model Assessment- Insulin Resistance (HOMA-IR) scores, and leptin and proinflammatory cytokines levels regardless of body weight [4].

In obese children, low-carbohydrate (L-CHO) diet may induce a better improvement of HOMA-IR and triglyceride levels compared to lipid restriction [5]; however, other reports have indicated that a low-fat (L-F) diet results in a better improvement of triglyceride levels [6].

For a better understanding of the effects of both types of macronutrient restriction, the impact on appetite-satiety mechanisms should be assessed. The Children’s Eating Behavior Questionnaire (CEBQ) is a validated tool to identify diverse signals related to satiety, enjoyment of food, response to different foods, and emotional components of appetite-satiety physiology in children [7].

In children food intake and attitudes toward meals, depend on maternal influences. Maternal feeding restriction is associated with a child’s food approach traits and responsiveness to food [8]; however, appetitive traits may respond differently to dietary restriction of carbohydrates or fats, and to our knowledge this subject has not been studied in children.

Leptin and fibroblast growth factor 21 (FGF21) are related to the regulation of food ingestion and energetic balance. Leptin is a key appetite-regulating hormone produced in the white adipose tissue that acts on the hypothalamus. Leptin suppresses food intake and increases energy expenditure by means of adrenergic stimulation [9] and stimulates peripheral fat oxidation and influences in glucose homeostasis by direct action on the pancreatic β cells [10]. Therefore, deficiency of leptin or its receptors facilitates obesity, increases insulin resistance and impairs glucose tolerance. In humans, a diet with fat [11] and carbohydrate [12] restriction decreases leptin levels.

FGF21 is a product of the liver and adipose tissue with important roles in food intake, and energy expenditure [13]. FGF21 mediates the metabolic response to starvation [14]. FGF21 enhances insulin sensitivity, decreases triglyceride concentrations, and ameliorates obesity-associated hyperglycemia and hyperlipidemia [15]. FGF21 also represses *de novo* lipogenesis by mediation of sterol regulatory element binding protein 1c transcription [16]. FGF21 is proposed as a mediator of glucagon regulation of glucose and lipid metabolism [17] and is an independent predictor of the metabolic syndrome in adults [18] but not in children [19].

In animal models, the effect of a L-CHO diet on FGF21 levels is controversial. In mice, a L-CHO diet increases as an adaptive response to malnutrition [20]; however, in another study FGF21 levels did not change [21]. In adults, overfeeding increases FGF21 levels [22], and an acute response was found with a fructose load [23].

In obese children who participated in exercise, behavior, and nutrition therapy over the course of 1 year, body mass index (BMI) diminution was accompanied by a decrease in FGF21 levels [24]; however, FGF21 did not change in adults on a ketogenic diet [25].

Surgical interventions in obese adults with intragastric balloon [26], laparoscopic sleeve gastrectomy [27] or gastric banding [28] decreases BMI and FGF21 levels; however in other studies, FGF21 levels did not change after intervention [29].

Carbohydrate or fat restrictions in the diet are strategies used to treat obesity with conflicting effects on appetite and satiety. Nevertheless, the effect on hormone and metabolic factors and the impact on long-term eating behavior are not well defined. Therefore, we examined the effect of restriction of carbohydrates or lipids, on diverse aspects of eating behavior and the metabolic and hormonal responses including leptin and FGF21 levels.

## Subjects and methods

In a prospective clinical intervention, we recruited 120 obese children (58 boys and 62 girls) with a BMI higher than the equivalent of 30 kg/m^2^ for an adult, as corrected for gender and age according to the International tables of Cole et al. [30]. The participants were 6 to 12 years old and were recruited from grammar schools from the León City at the region central of Mexico. The participants did not have clinical evidence of hypothyroidism, chronic infections, congenital or metabolic diseases. The nature and purpose of the study was explained to the children and their parents and the confidentiality of individual results was guaranteed. All of the parents who accepted their children’s participation signed informed consent forms. The study was approved by the Institutional Ethics Committee, in compliance with the Declaration of Helsinki.

### Data collection

All of the measurements were obtained at baseline and after the intervention. The children’s time of sleep (hours/day); exercise (minutes/week); and screen time, mainly spent on TV, computer and video games (minutes/week) were collected by direct questioning of the children and at least one of their parents. We collected the family history of obesity and diabetes as well as mother’s BMI.

Eating behavior scores were calculated using the CEBQ scale [7], as reported by one of the parents, which included 35 items to evaluate the following seven subscales: Satiety responsiveness/slowness in eating (nine items), Food fussiness (six items), Food responsiveness (five items), Enjoyment of food (four items), Desire to drink (three items), Emotional overeating (four items) and Emotional undereating (four items).

Weight and standing height were obtained while the children wore indoor clothing but no shoes using a roman type scale and a Harpenden stadiometer to determine the BMI value. The waist girth was measured while the children wore indoor clothes using a non-extendible flexible tape at the midpoint between the last rib and the iliac crest. The hip girth was measured at the point of maximum girth over the buttocks to obtain the waist-hip ratio (WHR). The skin fold thickness was obtained at bicipital, tricipital, suprailiac and subscapular sites to calculate body density as follows: For boys = 1.1533−(0.0643 × log ∑ (four measurements of skin fold thickness)), and for girls = 1.1369−(0.0598 × log ∑ (four measurements)). The percent body fat [31] was calculated as {4.95/body density - 4.5} × 100. The children were examined for pubertal development, scoring pubarche, adrenache, thelarche and menarche. The testicular volume was registered for the boys.

A venous blood sample was obtained after 12 h of fasting to measure hormones and metabolites. Serum glucose, triglycerides and total cholesterol concentrations were measured using enzymatic methods with a chemistry analyzer (Auto KEM II, Kontrollab, Italy). Serum insulin, leptin and adiponectin were analyzed by radioimmunoassay using a Millipore kit (St. Charles, Mo), with intra- and inter-assay variation coefficients of 4.4 and 6.0 % for insulin, 3.4 and 3.6 % for leptin, and 6.2 and 9.2 % for adiponectin. FGF21 was measured by ELISA, (Mediagnost, Reutlingen, Germany), with intra- and inter-assay variation coefficients of 3.5 and 5.2 %. Insulin resistance was estimated with HOMA-IR.

### Intervention

The children were randomly assigned to two different dietary treatments: a L-F diet and a L-CHO diet (60 children each) for a 2-month period. The diets were elaborated according to the recommendations of the American Academy of Pediatrics [32] and the Official Mexican Norm 043 (NOM-043-SSA2-2005). The L-CHO diet was 50 % carbohydrate, 30 % fat and 20 % protein, while the L-F diet with 60 % carbohydrate, 25 % fat and 15 % protein. The caloric intake was calculated individually based on total energy expenditure for each child according to age requirements [33]. Then, the distribution of macronutrients, depending on the assigned dietary treatment was calculated. The diet prescribed was explained to the parents, indicating the choice of foods and procedures of measurements, to be carried out at home. Deviations from the indications were assessed in the collection of the compliance evaluation.

### Statistical analysis

The data are shown as the means and standard deviations. The variables were tested for normality with the K-S test. Comparison of the data for both of the diet groups was carried out with the t-test for independent variables. Each group was compared before and after the dietary treatment using ANOVA for repeated measures, testing as covariates sex, age, pubarche and adrenarche.

Factors associated with FGF21, leptin levels and satiety were analyzed by multiple regression, with stepwise selection of significant variables. We used the same model for the analysis of serum FGF21 and leptin levels as dependent variables testing as candidate regressors BMI, percent body fat, age, sex, glucose, insulin, HOMA, total cholesterol, triglycerides, adiponectin, hours of sleep, exercise, screen time and the seven subscales of eating behavior. We analyzed the factors associated with satiety taking as candidate regressors BMI, age, sex, leptin, adiponectin, insulin, FGF21, time of exercise, screen time, and hours of sleep.

## Results

One hundred and twenty children were included in the study. The children had a mean age of 9.8 ± 1.6 years, a mean BMI of 28.1 ± 3.6 kg/m^2^, a mean WHR of 0.96 ± 0.05, and a mean percent body fat of 41.01 ± 3.46. Their basal dietary consumption was as follows: kilocalories 2161 ± 606, lipids 31.6 ± 6.3 % and carbohydrates 54.1 ± 7.4 %. Initially the children were randomized into two groups. The data from both of the groups had a normal distribution and were similar compared with a t-test for independent variables.

The 2 months follow-up was completed by 53 (88.3 %) subjects on the L-CHO diet and 45 (75 %) with on the L-F diet. The children decreased their total caloric intake by a mean of 2436 kJ/day (582 kcal) after 8 weeks (*p* < 0.00003). The group on the L-CHO diet decreased 2554 kJ/day (616 kcal), *t* = 6.14, *p* < 0.0000001. The group on the L-F diet decreased 2457 kJ/day (587 kcal), *t* = 7.50, *p* < 0.0000001 a value within the range of expected dietary compliance.

### Effect of low-carbohydrate diet

After 2 months on the L-CHO diet, the BMI, leptin and FGF21 levels decreased. An increase in total cholesterol was observed as a result of an increase in high density lipoprotein cholesterol (HDL) levels. No change was found for HOMA-IR. In regards to eating behavior, food responsiveness, enjoyment of food and emotional overeating decreased significantly, and satiety responsiveness increased marginally (Table 1).

**Table 1:** Effect of the low-carbohydrate diet analyzed with repeated measures ANOVA test

	Basal	Two months after the dietary intervention	f	p-value
	Mean ± SD	Mean ± SD		
	*N* = 53	*N* = 53		
BMI (kg/m^2^)	27.62 ± 3.28	26.41 ± 3.22	104.50	<0.0000001
WHR	0.97 ± 0.05	0.96 ± 0.05	2.82	0.09
Percent body fat	40.88 ± 3.11	40.18 ± 2.80	2.55	0.12
Glucose (mmol/l)	4.77 ± 0.46	4.78 ± 0.36	0.02	0.89
Triglycerides (mmol/l)	1.50 ± 0.52	1.43 ± 0.48	0.92	0.34
Total cholesterol (mmol/l)	3.76 ± 0.65	3.98 ± 0.66	6.30	<0.02
HDL, cholesterol (mmol/l)	1.07 ± 0.29	1.17 ± 0.18	8.20	<0.006
LDL, cholesterol (mmol/l)	2.01 ± 0.69	2.15 ± 0.59	2.47	0.12
HOMA	6.12 ± 3.59	5.59 ± 2.48	1.83	0.18
Leptin (ng/ml)	25.75 ± 12.06	17.03 ± 9.81	52.86	<0.0000001
Insulin (pmol/l)	197.52 ± 109.31	180.22 ± 72.02	1.75	0.19
Adiponectin (ng/ml)	14.83 ± 7.78	17.79 ± 10.24	2.83	0.09
FGF21 (pg/ml)	202.58 ± 115.27	117.15 ± 96.70	35.19	<0.0000001
Satiety responsiveness	10.75 ± 5.90	11.81 ± 5.28	3.76	0.06
Fussiness	11.85 ± 4.64	11.21 ± 4.78	1.33	0.25
Food responsiveness	11.23 ± 4.68	7.92 ± 5.18	39.69	<0.0000001
Enjoyment of food	11.87 ± 2.54	10.19 ± 2.72	13.61	<0.0006
Desire to drink	8.14 ± 3.33	7.23 ± 3.83	3.89	0.06
Emotional undereating	6.33 ± 3.09	5.94 ± 3.53	2.28	0.14
Emotional overeating	6.79 ± 3.74	5.96 ± 3.75	4.93	<0.03

*BMI* Body mass index, *WHR* Waist-Height Ratio, *HDL* High density lipoprotein, *LDL* Low density lipoprotein, *FGF21* Fibroblast growth factor 21

### Effect of low-fat diet

After 2 months on the L-F diet, the BMI, leptin levels, LDL-cholesterol and FGF21 levels decreased and HDL-cholesterol increased. Regarding eating behavior, food responsiveness and enjoyment of food decreased significantly and satiety responsiveness increased marginally (Table 2).

**Table 2:** Effect of the low-fat diet analyzed with a repeated measures ANOVA test

	Basal	Two months after the dietary intervention	f	p-value
	Mean ± SD	Mean ± SD		
	*N* = 45	*N* = 45		
BMI (kg/m^2^)	28.75 ± 4.11	27.54 ± 4.10	22.67	<0.00002
WHR	0.95 ± 0.15	0.97 ± 0.06	0.48	0.49
Percent body fat	41.11 ± 3.83	40.69 ± 2.87	0.99	0.33
Glucose (mmol/l)	4.65 ± 0.47	4.74 ± 0.34	1.23	0.27
Triglycerides (mmol/l)	1.47 ± 0.61	1.47 ± 0.60	0.001	0.97
Total cholesterol (mmol/l)	4.03 ± 0.78	3.89 ± 0.75	2.52	0.12
HDL-cholesterol (mmol/l)	1.02 ± 0.21	1.14 ± 0.19	20.11	<0.00006
LDL-cholesterol (mmol/l)	2.26 ± 0.77	2.08 ± 0.71	6.55	<0.01
HOMA	6.04 ± 3.00	6.06 ± 2.93	0.01	0.93
Leptin (ng/ml)	27.94 ± 10.02	19.96 ± 10.62	24.75	<0.00001
Insulin (pmol/l)	200.22 ± 93.34	199.88 ± 96.26	0.002	0.97
Adiponectin (ng/ml)	15.10 ± 6.90	16.77 ± 9.35	1.54	0.22
FGF21 (pg/ml)	152.10 ± 97.07	112.59 ± 96.91	5.47	<0.02
Satiety responsiveness	11.17 ± 5.91	12.64 ± 4.30	3.46	0.07
Fussiness	10.26 ± 5.03	10.98 ± 4.92	1.53	0.22
Food responsiveness	11.64 ± 5.13	8.86 ± 4.57	13.64	<0.0006
Enjoyment of food	12.24 ± 2.65	10.55 ± 2.56	13.12	<0.0008
Desire to drink	8.88 ± 3.06	8.43 ± 22.86	0.80	0.38
Emotional undereating	6.24 ± 3.11	5.83 ± 3.15	2.66	0.11
Emotional overeating	6.88 ± 3.51	6.26 ± 3.19	1.48	0.23

*BMI* Body mass index, *WHR* Waist-Height Ratio, *HDL* High density lipoprotein, *LDL* Low density lipoprotein, *FGF21* Fibroblast growth factor 21

### Comparison of changes between both dietary interventions

Table 3 compares the effect of both diets. The total and LDL-cholesterol levels significantly decreased with the L-F diet. A similar feeding behavior was observed in both of the groups.

**Table 3:** Comparative effect of both dietary interventions with t test for independent variables

	L-CHO diet	L-F diet	t-value	p-value
	Mean ± SD	Mean ± SD		
	*N* = 53	*N* = 45		
Δ BMI (kg/m^2^)	−1.21 ± 0.86	−1.21 ± 1.70	0.02	0.99
Δ WHR	−0.01 ± 0.04	0.02 ± 0.14	1.20	0.23
Δ Percent body fat	−0.70 ± 3.17	−0.42 ± 2.85	0.45	0.66
Δ Glucose (mmol/l)	0.01 ± 0.38	0.09 ± 0.53	0.90	0.37
Δ Triglycerides (mmol/l)	−0.07 ± 0.50	0.004 ± 0.61	0.61	0.54
Δ Total cholesterol (mmol/l)	0.22 ± 0.63	−0.14 ± 0.56	−2.86	<0.005
Δ HDL, cholesterol (mmol/l)	0.10 ± 0.26	0.12 ± 0.17	0.28	0.78
Δ LDL, cholesterol (mmol/l)	0.14 ± 0.67	−0.18 ± 0.68	2.33	<0.02
Δ HOMA	−0.54 ± 2.85	0.02 ± 1.75	1.11	0.27
Δ Leptin (ng/ml)	−8.72 ± 8.82	−7.98 ± 10.40	0.38	0.71
Δ Insulin (pmol/l)	−17.29 ± 94.31	−0.35 ± 58.62	1.01	0.31
Δ Adiponectin (ng/ml)	2.96 ± 12.67	1.67 ± 8.69	−0.56	0.58
Δ FGF21 (pg/ml)	−85.42 ± 94.43	−51.67 ± 69.17	−1.68	0.09
Δ Satiety responsiveness	1.06 ± 3.93	1.48 ± 5.14	0.45	0.66
Δ Fussiness	−0.64 ± 3.96	0.71 ± 3.73	1.68	0.10
Δ Food responsiveness	−3.31 ± 3.79	−2.79 ± 4.89	0.58	0.56
Δ Enjoyment of food	−1.67 ± 3.27	−1.69 ± 3.02	−0.03	0.98
Δ Desire to drink	−0.90 ± 3.30	−0.45 ± 3.28	0.66	0.51
Δ Emotional undereating	−0.39 ± 1.84	−0.41 ± 1.61	−0.06	0.96
Δ Emotional overeating	−0.83 ± 2.68	−0.62 ± 3.30	0.34	0.74

*L-CHO* Low-carbohydrate diet, *L-F* Low-fat diet, *BMI* Body mass index, *WHR* Waist-Height Ratio, *HDL* High density lipoprotein, *LDL* Low density lipoprotein, *FGF21* Fibroblast growth factor 21

### Factors associated with the FGF21 levels

Using the multiple regression procedure, we found that before the diet, FGF21 was positively associated with total cholesterol, triglycerides and BMI. After the L-CHO and L-F diets FGF21 remained associated only with triglyceride levels (Table 4).

**Table 4:** Factors associated with FGF21, leptin and satiety analyzed by multiple regression

Dependent variable		BETA ± SE	t	p-value
FGF-21 - Before treatment	Total cholesterol	0.28 ± 0.11	2.56	<0.01
	Triglycerides	0.31 ± 0.11	2.84	<0.006
	BMI	0.30 ± 0.11	2.79	<0.007
FGF-21 - After L-CHO diet	Triglycerides	0.42 ± 0.14	2.93	<0.005
FGF-21 - After L-F diet	Triglycerides	0.43 ± 0.17	2.62	<0.01
Leptin - Before treatment	BMI	0.60 ± 0.09	6.32	<0.0000001
Leptin - After L-CHO diet	BMI	0.74 ± 0.10	7.70	<0.0000001
Leptin - After L-F diet	BMI	0.47 ± 0.14	3.42	<0.001
Satiety - Before treatment	Screen time	−0.19 ± 0.09	−2.11	<0.04
	Insulin	−0.17 ± 0.09	−1.90	<0.05
Satiety - After L-CHO diet	No variable included			
Satiety – After L-F diet	No variable included			

*BMI* Body mass index, *FGF21* Fibroblast growth factor 21, *L-F* Low fat diet, *L-CHO* Low carbohydrate diet

Considering this association we examined the possible relationship of FGF21 with pubertal development. ANOVA for adrenarche stages in both sexes did not show an association with FGF21 (*f* = 0.005, *p* = 0.94). With univariate regression analysis for young boys, testicular volume was not associated with FGF21 (*p* = 0.22).

### Factors associated with leptin

For the basal study, the only factor associated with leptin was BMI. After dietary treatment with the L-CHO and L-F diets, the association with BMI remained significant (Table 4).

### Factors associated with satiety

Before the treatment satiety was negatively associated with screen time and insulin levels. After the treatment no factor was associated (Table 4).

### Influence of age on the response to macronutrient restriction

In the multiple regression analysis for hormones and satiety, gender and age were not included in the model. In an additional analysis we divided both of the groups by ages 6 to 10 (*n* = 76), and >10 to 12 years (*n* = 22). In the 6 to 10 years of age group, the factors associated with leptin, FGF21 and satiety were the same as in the total group (Table 4); however in the >10 to 12 years group, leptin was associated with BMI before intervention, and after either diet. No associations were found with FGF21 or satiety. This finding indicates that in our group early pubertal activation is not adequately represented to show its influence on satiety or hormone changes.

## Discussion

A 2-month intervention may be limited by tolerance. A prolonged intervention may permit a further reduction of obesity. Adult obese people on a diet may have serious rebound effects. Whether problems of treatment in adults may be more manageable in children is important to clarify.

The balance of macronutrients in the design of dietary restriction for obesity in children is an important subject. To solve this point, the impact of different diet schemes on eating behavior, hormones and metabolic response must be studied in diverse populations. In adults, the comparison of carbohydrate or fat restriction during 8 weeks did not show differences in satiety [34]. Yet, another study showed a lower perception of hunger in the group on a L-CHO diet after 6 weeks [35].

Nonetheless in obese children, differences in feeding behavior, under fat or carbohydrate restriction have not been reported. In this study we found that changes in appetite traits were not different with carbohydrate or fat restriction. After 2 months on either diet, food responsiveness and enjoyment of food decreased, and satiety increased marginally, probably because of reduced feeding pleasure. Our results indicate that eating behavior in children is modified favorably with both diets. Emotional overeating decreased in a marginal manner only under the L-CHO diet. There is no previous information about this subject. An appetite adaptation to macronutrient restriction occurred for the children after the 2-month study and further supports the continuation of the dietary program. The decrease in appetite may also correlate with the bland diets.

Before the intervention, satiety was negatively associated with screen time and insulin levels. Previous reports showed that in children, a long time spent before a screen increases caloric intake by delaying normal mealtime satiation [36]. This finding supports the concept that entertainment before a screen may increase obesogenic behavior. A recent study in adults reported that satiety signals are related to ghrelin and triglycerides but not to insulin levels [37]. Nevertheless, no previous information about insulin and satiety in children exists, and therefore, this subject should be analyzed in future studies.

In our study LDL-cholesterol and total cholesterol had a greater decrease with the L-F diet. In adults the information about this subject is controversial because one report shows that a L-CHO diet for 8 weeks decreased LDL-cholesterol levels, showing a less atherogenic lipid profile compared to the L-F diet [34]; however, another study reported that both restriction diets induced a similar decrease of LDL-cholesterol [38]. HDL-cholesterol increased after both interventions in accordance with a previous report in adults [39].

We found that leptin decreases with both diets, and this hormone was positively associated with BMI. Leptin release is dependent on both adipose mass and nutrient flux, which is mainly represented by hexosamines in the adipocyte [40]; however, satiety increased marginally, and we must consider that appetite and satiety result from the interplay of several factors, included ghrelin and nutrient signaling [41] and factors related with leptin resistance [42].

Insulin levels showed a trend to a non-significant decrease. In adults, short-term food restriction induces a decrease in insulin levels [43]. In children, a significant insulin decrease has been reported with only 6-month [2] and 16-week diets [44]; however, in children insulin diminution may be observed after 2 weeks of diet and exercise [4]. This finding may indicate that in children, leptin is more sensitive to calorie restriction than insulin levels.

FGF21, which is proposed to be an important hormone for metabolic adaptation to starvation and food restriction [14], decreased with both diets. In humans, the decrease rather than the increase of FGF21 levels with dietary restriction is not congruent with that concept. This finding is in agreement with previous studies with food restriction in children [24] and adults [28]. Our findings are of interest because a similar response was found with the L-CHO and L-F diets. We are not aware of similar previous observations.

Before the diet, FGF21 was associated with BMI, total cholesterol and triglycerides. In regards to triglyceride levels, this association is in agreement with previous reports [45] and in accordance with the concept that FGF21 is a biomarker of metabolic conditions. In adults, the circulating levels of FGF21 increase under adverse lipid profiles, obesity, metabolic syndrome, impaired glucose tolerance, type 2 diabetes mellitus, or atherosclerosis [18], but in children its relationship with metabolic syndrome has not been confirmed [19].

Interestingly, the association of FGF21 with triglyceride levels remained after both diets. Transgenic mice with an overexpression of FGF21 have a reduction in plasma triglyceride levels [14]; however, in human subjects, FGF21 correlates positively with triglycerides suggesting that FGF21 is a result of obesity and may compensate for the metabolic stress in states of obesity and insulin resistance. Considering that the administration of FGF21 has an antilipolytic effect, the positive association between FGF21 and triglycerides levels may indicate a state of FGF21 resistance as proposed by Fisher et al. [46] in which FGF21 fails to maintain triglyceride levels within the normal range. FGF21 may act as a compensatory signal to mitigate metabolic stresses due to obesity [47].

Fgf21-null mice showed increased hepatic endoplasmic reticulum (ER) stress. Exposure of primary hepatocytes to palmitic acid elevated the mRNA levels encoding DNA damage-inducible transcript 3, an indicator of ER stress, and FGF21 in a peroxisome proliferator activated receptor alpha (PPARα) -independent manner. This finding suggests that lipid-induced ER stress can enhance hepatic FGF21 expression [48].

A closer examination of the results suggests that certain parameters trended down or up and were close to statistical significance, and thus, much larger cohorts need to be analyzed.

## Conclusions

In conclusion, to our knowledge this is the first study to compare the effect of carbohydrate or lipid restriction on appetite traits in obese children. Both types of dietary restriction improved the metabolic parameters. Leptin and FGF21 decreased with both diets. Leptin was correlated with BMI and FGF21 was associated with triglycerides, total cholesterol and BMI, but after the L-CHO and L-F diets, only triglycerides remained associated with FGF21. Before the diet, satiety was associated with less screen time, but after the diet, appetite traits were not associated with life-style, hormonal or metabolic factors. In this study we did not find important behavioral, metabolic or hormonal differences between both types of diets. Further studies are required to properly identify changes induced with different amounts of macronutrients.

## Abbreviations

LDL: Low density lipoprotein
HOMA-IR: Homeostatic Model Assessment- Insulin Resistance
CEBQ: Children’s eating behavior questionnaire
FGF21: Fibroblast growth factor 21
BMI: Body mass index
WHR: Waist-hip ratio
L-F: Low-fat diet
L-CHO: Low-carbohydrate diet
HDL: High density lipoprotein
ER: Endoplasmic reticulum
